# A new intervention to prevent social isolation in people with complex communication needs

**DOI:** 10.1038/s41598-024-63519-5

**Published:** 2024-06-03

**Authors:** João Canossa Dias, Ana Mineiro, Saskia Damen

**Affiliations:** 1 Department of Rehabilitation and Inclusion, ARCIL, 3200-065 Lousã, Portugal; 2 Institute of Health Sciences, Portuguese Catholic University, 1649-023 Lisboa, Portugal; 3 Center for Interdisciplinary Research in Health (CIIS), 1649-023 Lisboa, Portugal; 4 Faculty of Behavioural and Social Sciences, University of Groningen, Groningen, 9700 AB The Netherlands

**Keywords:** Health care, Medical research

## Abstract

While implementing communication interventions, practitioners follow diverse theoretical models. Different conceptual orientations influence the way professionals embrace the subject of communication and its disorders. This research project explores the co-creation and validation of a new model and intervention program to analyze and improve communication between persons with Complex Communication Needs and their caregivers. The methodology incorporated a comprehensive narrative review, as foundation for the new model and intervention proposal. Succeeding this stage, the team implemented an online Delphi Panel to improve and validate these results, involving 17 international renowned experts. Following the Appropriateness Method, 25 indications were subject to scrutiny and rated as appropriate with minimal values of disagreement among the evaluators. Qualitative feedback was used to improve the research products. Quality assurance measures were taken to ensure quality and transparency of the results. A new conceptual framework of atypical interpersonal communication and intervention program result from the investigation. The new model is inspired by the Transactional model and principles of Dialogism. The intervention consists of consultations with caregivers, using video analysis and a dialogical methodology to enhance communication. The next research phase is to pilot-test the intervention program with clinicians supporting persons with disability at risk of social isolation.

## Introduction

To define communication clearly and completely seems to be impossible and, probably, a never-ending endeavor^1^. The existence of various conceptualizations should be recognized as inevitable and natural, though it should not be considered inconsequential. The adoption of distinctive points of view will guide clinicians towards different directions, emphasizing certain aspects of communication in detriment of others^2^. Whilst intervening in the field of communication, professionals may adopt one of the three main “theoretical categories” through which the process of human communication may be analyzed^3^:

(i) Communication as a unidirectional process from sender to receiver;
(ii) Communication as a two-way process of meaning construction between communicators;
(iii) Communication as an omnidirectional and diachronic meaning negotiation between partners.

**Figure 1:**
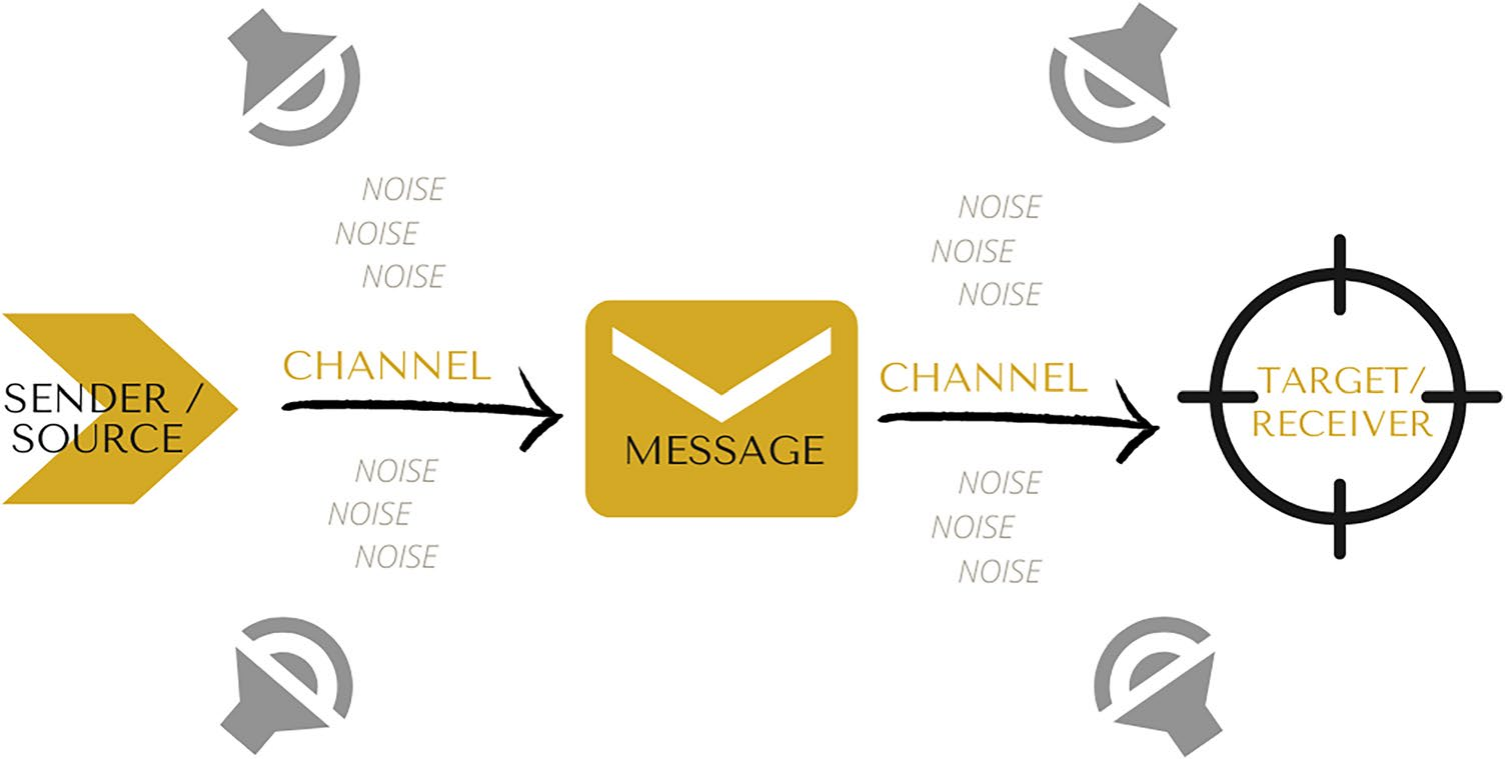
Original illustration of the Linear Model of Communication prepared by the authors, based in the literature review^4^, using the software Canva.

Following the first perspective is the Linear Model of communication, according to which the sender creates and encodes a message, sending it through a channel to a receiver. In order share the intended information, the sender uses a code shared with the receiver. This entity decodes the information, while dealing with distractions that disrupt transmission, in this model identified as noise^4^. Noise within the process interferes with the success of communication, impacting the way messages are sent and received, and potentially creating failure^5^. Below, in Fig. 1, the Linear Model of Communication is graphically illustrated.

**Figure 2:**
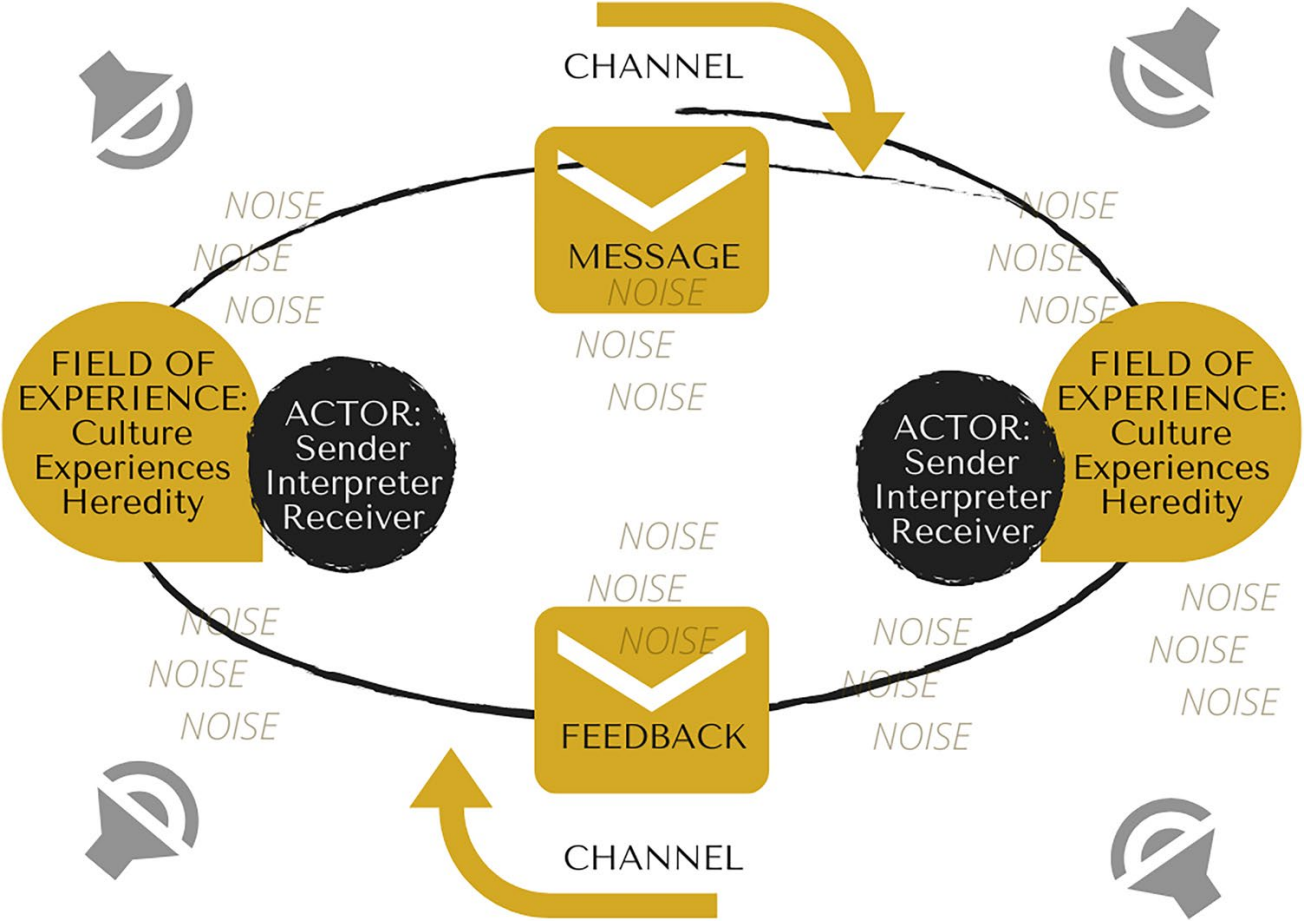
Original illustration of the Interactional Model of Communication prepared by the authors, based in the literature review^5^, using the software Canva.

Though the Linear Model of communication may represent specific communicate contexts (e.g., mass communication), it is hardly representative of the dynamic and complex nature of interpersonal communication, calling for a supplementary theoretical approach to analyze this phenomenon. Theories understanding communication as a two-way process are framed within the Interactional Model of Communication, valuing how actors engage in conversations and converge while sharing information^3^. Here, communication is conceptualized as an ongoing flow in two directions—from sender to receiver and from receiver to sender—in opposition to how it was represented in the linear perspective. A communicator can perform the role of either sender or receiver in the interaction, but never simultaneously. Another element that is crucial to the interactional conceptualization is feedback, the response to a message, which takes place after the message is received. One additional feature of this model is that each actor in communication presents with an individual field of experience—i.e., the person’s culture, experiences, and heredity—that influences the ability to communicate with others. Noise is present in the interactional context and may inhibit the effectiveness of communication^5^. The presented illustration—Fig. 2—represents the interactional understanding of the communicative exchange.

**Figure 3:**
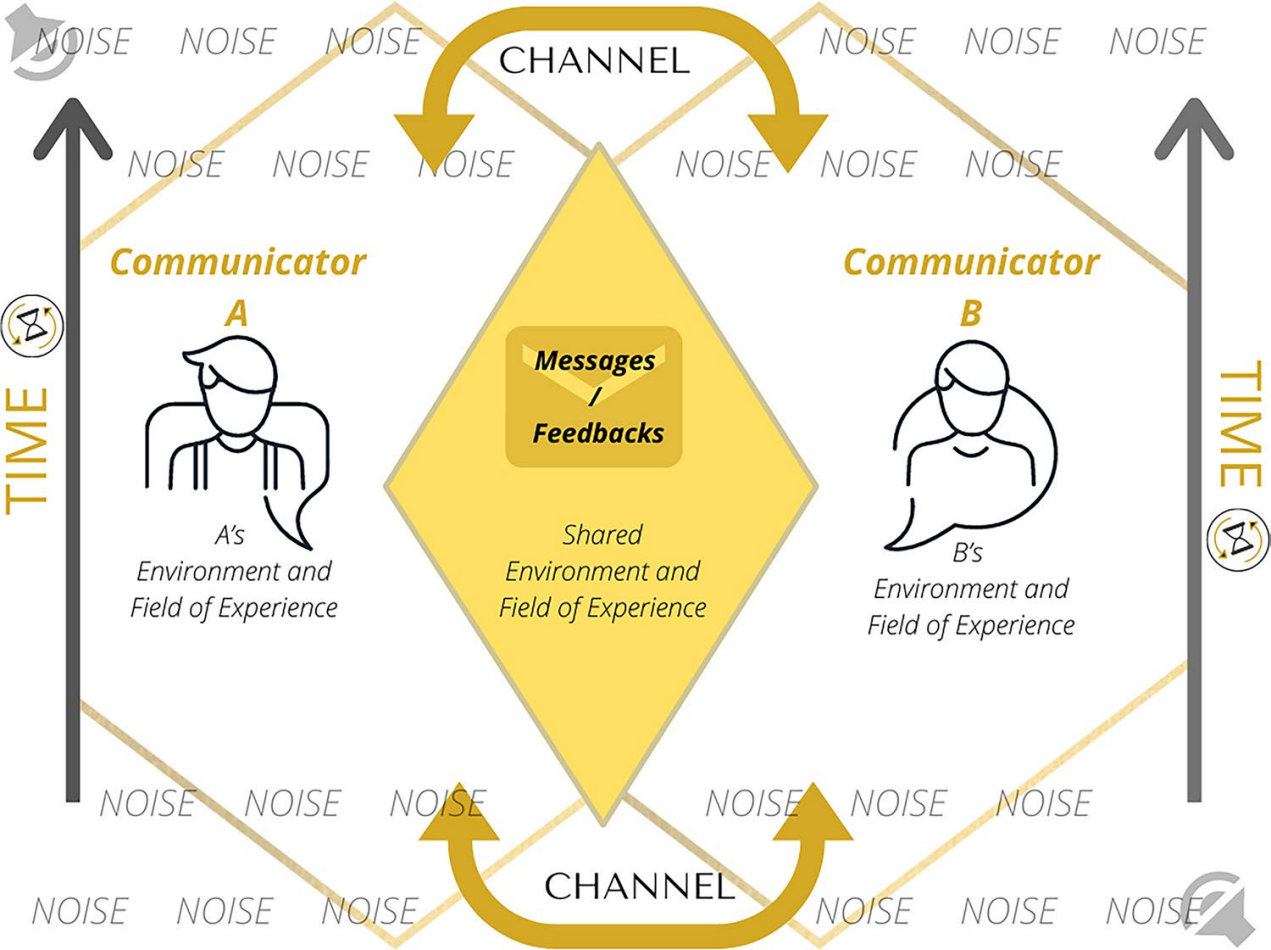
Original illustration of the Transactional Model of Communication prepared by the authors, based in the literature review^4,5^, using the software Canva.

An interactional understanding of the communicative process represents more closely how in-person communication between humans may function (e.g., in communicative exchanges mediated by technology), although it does not represent completely the synchronized process of two individuals interacting with each other, occupying simultaneously the roles of sender, receiver and interpreter. The Transactional Model assumes this perspective of communication as an omnidirectional and diachronic process of meaning negotiation. Interaction between partners plays a fundamental role in this multidirectional process of meaning co-creation^3^, implying that the communicators are focused on the ongoing interaction and meaning making over time, instead of concentrating on the transmission of individual messages between each other. According to this lens, participants send and receive messages and feedback simultaneously and not in a unidirectional or back-and-forth modality^4^. In cooperation, all participants are responsible for the effectiveness of the interaction and, not only do the communicators influence each other, it is assumed that messages are interdependent and sequential, with the influence of one message over the others^5^. As partners communicate, their separate fields of experience tend to merge, in an active effort for mutual understanding and co-construction of shared meaning^5^. A broad notion of noise embraces the existence of different types of distorting variables in the exchange^4^ and misunderstandings are seen as more than the interference of noise in the chain of messages/feedback; individuals should incorporate where the other in the relationship is coming from, in order to build shared meaning, and misunderstandings often rise when the partners have difficulty in doing so^5^. One last feature of the transactional archetype should be highlighted: transactional communication is considered diachronic. Instead of focusing on the transmission of messages/feedback, in a linear or circular way, the transactional paradigm brings the attention to growing interaction between communicators, developing over time^3^. The relevance of time is anchored in Dance’s Helical Model of Communication^6^, which explains that communicators improve their messages/feedback with several trials; whenever one communicates, the subject expands his abilities and the circles of communication grow continuously, similarly to the geometry of a helix, with increasingly wider circles. A visual representation of the Transactional Model of communication is presented as Fig. 3.

The Transactional Model of Communication appears to be the conceptual framework that better systematizes the complexity of interpersonal communication. Aligned with this model, Karen Bunning’s defines communication as a process between two or more persons, who are “working together”, coordinating their actions and reactions in response to each other and to the context^7^. Interestingly, Pearson, Nelson, Titsworth and Hosek^8^ embrace the idea of communication as a “project”, stating that “*communication is considered a process because it is an activity, an exchange or a set of behaviours—not an immutable product”* (p.8–9). Embedded in the Dialogical philosophy, Linell^9^ asserts that communication ought to be acknowledged in the form of communicative projects, described as “*other-oriented and jointly accomplished communicative actions, typically but not necessarily carried out in external interpersonal interaction*” (p. 178). The projects assume the form of two or more communicators interacting over sequences of acts, looking to establish a communicative fact that is mutually understood. These viewpoints emphasize the co-constructive nature of the communicative process (i.e., meaning-making and negotiation between communicators), and the first^7^ argues that its success depends on both sides—the “speaker” and the “listener”—and highlights the importance of the context for communication to be effective; in fact, part of the meaning comes from the context where the interaction takes place.

Having defined communication, in the context of interpersonal exchanges, it matters to investigate the other dimension of this process: communication disorders or miscommunication. Communication disorders are broadly defined by the American-Speech-Language-Hearing Association (ASHA) as impairments in “*the ability to receive, send, process, and comprehend concepts or verbal, nonverbal and graphic symbol systems*”, ranging in severity from mild to profound. A communication disorder may be developmental or acquired and may result in a primary disability or be secondary to other disabling condition^10^. From the point of view of Dialogism, difficulties in communication may be understood in a broader perspective, regarded as miscommunication. In this case, difficulties in communication are collectively and reciprocally generated, frequently because of misinterpretations of the partners’ intentions and related to different fields of experience^11^. Pondering the adoption of a transactional understanding of interpersonal communication, the expression Complex Communication Needs (CCN) may bring a more comprehensive understanding of this phenomenon, meaning that some individuals may not demonstrate all the communication skills required to fulfill their needs in different contexts^12^. This may happen because they do not use speech to communicate functionally, as a result of some kind of disability, relying on alternative forms of communication, such as gestures or graphic symbols^13^. It may also be the case that some of these people may be unintelligible to unfamiliar partners and/or they may struggle understanding the way others communicate^12^.

Although CCN tend to be linked to an individuals’ developmental or acquired disability^12^, the skills, sensitivity, patience, and honesty of their partners may have an enormous impact in the success or failure of the communicative exchanges^14^. According to Iacono^15^ (p.83), “*as the complexity of the disability increases, so does the complexity of communication needs and of finding an alternative system*” for the person to be an active participant in communication. The author goes further, explaining that “*people with disabilities must also rely on the skills of others to help them to be a part of a conversation*”, reinforcing the idea that communication is a two-way process, and, for the same reason, it should be seen as a two-way effort. As Dias^16^ concludes from a Grounded Theory study with caregivers of persons with complex disability, CCN may be present, but communication may certainly be successful, rich and diverse, depending on how supportive the communication context will be. There are, however, descriptions from persons with disability explaining that frequently partners without disability tend to dominate communication exchanges and limit the participation of the partner with CCN, by asking too many questions, occupying the majority of the conversational turns, providing few opportunities for the use of alternative systems of communication, interrupting frequently, and not always confirming the content of shared messages^17^.

Considering how impactful a disability may be in the person’s communication profile, one should not be surprised that many persons with complex disabilities are at risk of being socially isolated. In 2008, Sheridan Forster^18^ stated that the scarce research available on the topic indicated some of these people would only benefit from few minutes of social interaction daily, at the school or day service they would spend their time in. Recent investigation^19^ reinforces this finding, reporting that adults with disability experience social isolation and loneliness at significantly higher rates than those without disability, with damaging impact to their well-being. Looking at the situation of children with Neurodevelopmental Disorders (NDD) in specific, it was found that loneliness was related to negative consequences in terms of mental health, behavior, and emotional development, with long-term effect into their adult life^20^. According to the same authors, the lack of research on the topic of loneliness and children with NDD reveals that this issue is not yet considered to be of significant matter. There is, nonetheless, research^21^ reinforcing how critical it is to address the communication needs of persons with disabilities at an early age, considering the negative impact that may exist not only for their social participation, but also in terms of language, cognitive and literacy development, access to education, and, most of all, overall quality of life.

In a research project^22^ aiming to make information available to support improvements in best practices in communication with people with the most complex needs, several approaches to improve communication exchanges were highlighted, namely:

- Capturing and sharing fundamental information, using Communication Passports;
- Implementing formal approaches such as Intensive Interaction, Music Therapy, use of symbols, narrative approaches …;
- Implementing informal strategies; and
- Staff training.

Bearing in mind the relevance of communication partners in the success (or failure) of communicative exchanges, it matters to explore training approaches that enhance the skills of caregivers as communication agents. In a study about partners of adults with profound disability, Hanley, Dalton, Martin and Lehane^23^ concluded that the attitude, personal characteristics, familiarity with the other person and knowledge about alternative forms of communication were factors that could enhance or hinder communication exchanges. Their review provided important insights on the need to clarify the role and responsibilities of communication partners of persons with severe disability, reinforced the role of specialized professionals—i.e., Speech and Language Therapists (SLT’s)—and made evident the professionals’ need of additional training to better support the partners of communicators with the more complex needs. Bortoli and collaborators^24^ explore the perception of SLT’s regarding the implementation of communication intervention with students with multiple and severe disability, receiving feedback from research participants about the high level of expertise required for this kind of work. The required level of skill appears to develop over time, with ongoing professional development and opportunities to access formal education to update skills and knowledge and ensure evidence-based practices.

Research^24^ (p. 66) points in the direction of complexity when it comes to understanding communication processes and intervention with pupils with severe disability. According to the Complexity Theory, communication may be explained as “*a complex dynamic process that is embedded within not only individuals’ characteristics but also broader and equally complex contexts or systems such as institutions and cultures*”. Even though the research refers to other studies in the field of Speech and Language Therapy where the complexity paradigm was approached, to date it had not been applied to the understanding of communication intervention with students with multiple and severe disabilities. This finding is in line with Simmons and Watson’s work^25^, who have concluded that the dominant conceptual frameworks utilized to comprehend persons with the most complex disability are tendentially simplistic, reductive, and objectifying, overlooking how complex and dynamic the lifeworld of this population may be. On the other hand, embracing the complexity of communication with persons with the more complex disabilities, following theoretical views that flow in that direction, may have beneficial results in how clinicians perceive this phenomenon and positive impact on the intervention procedures. In her essay on the topic, Nafstad^26^ concludes: *“communication as viewed in terms of dialogical theory can help professional carers overcome the mainstream idea that it takes conventional linguistic skills to overcome the pain of the isolation”.*

Reflecting upon the reviewed literature, and aligned with the authors’ professional experiences, the researchers identified the urge to develop a project to design an intervention approach to work with caregivers of persons with CCN, embracing how complex the process of interpersonal communication really is. For this reason, the conceptualization and implementation of such intervention should be grounded in a Transactional Model of communication and instilled by principles of Dialogism; such intervention was not found to exist to date. Bearing this in mind, the researchers considered that co-creating a new model and intervention program starting from the best evidence available from the literature, combined with contributions from highly specialized professionals, would correspond to the team’s research objectives. These are, as followed:

- To co-create and validate a theoretical model to illustrate and explain the complex process of interpersonal communication with individuals with CCN, following the Transactional Model of communication and principles of Dialogism;
- To co-create and validate an intervention program, informed by the newly established theoretical model, to support professionals in their work with communication partners of persons with complex communication needs.

By doing so, the expectation is to create resources to support communication specialists in their interventions and having a positive impact in the lives of persons with CCN.

## Methods

The research methodology incorporated a two-stages process to co-create a new model and intervention program and to improve and validate these results, based on relevant literature and incorporating input from specialists in the field. The first step consisted of a thorough literature review on the topics of communication sciences and CCN. Succeeding this stage, the research team engaged in a panel with international experts to improve and validate the new model and intervention program.

### Narrative literature review: development of the new model and intervention program

To achieve the goal of co-creating a novel model and intervention proposal, the researchers invested in an extensive literature review on the topic of communication sciences, focusing on interpersonal communication. In clinical research, reviews are useful when developing practice guidelines, summarizing relevant theoretical knowledge on the topic under study^27^. From the two main options, systematic or non-systematic revision^27^, the team opted for the second one. As the name indicates, non-systematic or narrative reviews do not follow a systematic procedure, weaving together important literature based on the exposure, expertise, and experience of the authors^28^. Restricting the focus on a well-defined subject and defining clear inclusion criteria for the literature search^27^ were strategies utilized to ensure quality of the review work. Literature was selected during the third quarter of 2020, considering the criteria of being books or scientific papers published within the previous ten years (2010-2020). According to the authors’ judgement, publications representing meaningful perspectives published before 2010 were also considered, given its “historical” importance to understand communication theory and models.

In the co-creation of the theoretical model, the corpus of research suggested three main theoretical “inclinations” that would serve the purpose of explaining communication between caregivers and individuals with complex communication needs. The Transactional Model of Communication illustrates the multidirectional, diachronic, and continuous-in-time nature of interpersonal communication^3^. Dialogism, as a philosophical stance, argues that it is impossible to effectively understand communication, if communication acts are analyzed in isolation from its sequence and disconnected from the context; there is a contingent and co-creative feature in communication exchanges, justifying a dialogical conceptualization of interactions in the form of communicative projects^9,11,29^. Lastly, the complexity approach would enable the exploration of the multifaceted interactions between variables at different levels, and how these influence each other during communication processes with persons with severe disability^24^.

A grounded theory research^16^, previously developed by the first author, analyzed communication exchanges between persons with atypical communication profiles, due to a disability or NDD, and their caregivers. This research provided the nine fundamental categories of atypical interpersonal communication that served as basis for the first version of the new model: (i) sensory information, (ii) emotional coregulation, (iii) dyadic/triadic foci of attention, (iv) active participation in shared activities, (v) communicative initiations, (vi) balanced exchange of communicative turns, (vii) communicative intentionality, (viii) developmental proximity and support and (ix) meaning negotiation and co-creation. Following the three mentioned theoretical pillars of Transactionality, Dialogism and Complexity, the researchers reorganized these nine components into the Complex of Continuous Communication (CCC). As the CCC graphical representation was finalized and its narrative explanation written, this part of the research was submitted and accepted for publication in a peer-reviewed scientific journal within the field of philosophical sciences^30^, reinforcing its adequateness as theoretical framework.

Inspired by the CCC model, the researchers started developing the intervention proposal to support communication specialists in their clinical intervention with caregivers of persons with CCN. Three existing and validated intervention programs were thoroughly explored and guided the creation of the new suggestion:

- The *Intensive Interaction* approach^31,32^, considering its focus on improving the Fundamental Principles of Communication with persons with severe disability;
- The *HOP—Hanging Out Program*^18^, and its Portuguese version *ToP—Programa Tempo Partilhado*^33^, which advocates for caregivers to intentionally and regularly spend time and fully dedicate their attention to the communicative exchange with their partners with disability;
- The *Video Interaction Guidance*^34^, which focuses on video analysis to promote positive changes on the relationship between dyads, favoring communication and reinforcing the support provided to caregivers.

The mentioned intervention methods, along with others explored during the literature review, were important sources of information and inspiration. However, these approaches did not provide the professional with a specific theoretical model of atypical interpersonal communication based in the transactional and dialogical perspectives, such as the CCC model, or have the Interactional Model of communication theoretical foundation; for this reason, the development of a program based on the CCC model was seen as advantageous The intervention program was prepared, including a descriptive explanation of all the processes and procedures and a set of templates to serve as supporting documents for its implementation. Since the theoretical background was the CCC model, the intervention proposed was entitled Program 3^C^.

### Panel of experts: improvement and validation of the CCC model and of the program 3^C^

Once the proposals for the theoretical model and intervention program were finalized, the researchers proceeded to its improvement and validation using a panel of experts (PE) methodology and following the Delphi Method (DM). This research design has gained popularity and acceptance within the scientific community, as a method to reach consensus in deciding on the appropriate lines of action, particularly in educational and health research^35^. It generally involves assembling groups of experts who reply to several rounds, responding to specific questions and aiming to reduce dispersion and reach consensus on best-practices^35^. Even though the approach does not allow for the generation of large-samples quantitative data, it delivers a scientific methodology suited to complex and multifaceted issues that benefit from subject matter experts’ insight^36^.

Being the authors’ intention to include international experts, a modified online version of the DM was considered as an adequate procedure. The work of Khodyakov and colleagues^37^ validated this option, reiterating that the online DM may be used to involve stakeholders in different moments, allowing for greater scalability, reducing costs, and enhancing the participation of different specialists. The specific contours of the PE were defined according to the RAND/UCLA user’s manual^38^, the original guide to the Appropriateness Method that specifies every step of the process. The authors of this approach believe that combining the best available scientific evidence (through the presentation of results of a literature review) with the collective judgement of experts (via the discussion with a panel) would be effective in providing a statement regarding the appropriateness of performing specific procedures at the level of patient-specific needs^38^. Posterior work, within the clinical setting^39,40^, validated the use of RAND’s Appropriateness Method, for its successful application in consulting professionals to inform the development of professional guidance and achieving evidence-based recommendations. Following the method, two rounds were necessary to achieve consensus and rate the appropriateness of the CCC model and Program 3^C^.

According to the Appropriateness Method, the number of panelists should be from seven to fifteen, with the “magical” number of nine being referred in the literature. Some variation around this size is acceptable, since it brings to the panel enough cognitive diversity, while being small enough to allow everyone to be involved in the discussions^38^. On the look for different perspectives, the research team invested in the dissemination of a Call for Experts using an Invitation Letter (IL) distributed mostly via e-mail, social networks and asking support to representative national (e.g., Portuguese Society of Speech and Language Therapy, Higher Educations Institutions in the field of Health Sciences) and international organizations (e.g., Communication Matters, International Society for Augmentative and Alternative Communication) in the field of CCN and communication intervention. Moreover, the main researcher took part in research events connected to the topic, *fora* where potential experts would be present. Criteria for including experts from the *Academia* was defined—(i) holding a PhD degree and (ii) being involved in research projects on the topic of CCN within the last five years. To include the perspective of those in the field, experts from the professional practice were also selected, following the specialist criteria from the Portuguese legislation^41^—(i) having ten or more years of field experience and (ii) holding a relevant *Curriculum Vitae* in the field of expertise. As spontaneous applications from experts were received, additional information about the panel process was sent to each candidate. In case of fulfillment of the inclusion criteria, the administrative requirements were the collection of socio-demographic information to characterize the group of participants and the signing of an Informed Consent Form and Non-Disclosure Agreement.

For the first round, a presentation of the CCC Model and of the Program 3^C^ were organized using Microsoft PowerPoint, and shared with the experts, as well as PDF files of the eight templates needed for the intervention. A Review Questionnaire (RQ) was used to support the analysis, composed of 25 questions, each referring to a concept/model, explanation, instruction, process, template, or organizational aspect of the program. Instructed by the Appropriateness Method^38^, a nine-points Likert scale—with one (Completely Inadequate) being the minimum level of adequacy, and nine (Completely Adequate) being the maximum—was used, along with blank boxes to collect the experts’ supplementary qualitative input. The reasonable period of four weeks was defined as deadline for the first analysis.

Prior to round number one, all the materials were subjected to a pilot-test^37^ with four respondents that were excluded from the sample, from which several improvements were implemented. It should be noted that all documentation used and material for analysis was linguistically reviewed by a professional specialized in the working language (English) and a native speaker of the same language, to avoid interferences of any linguistic imprecision in the analysis. As the best-practices^37,38^ recommended, different strategies were used to facilitate communication between researcher and experts, specifically: integration of explanatory videos within the materials prepared for analysis; follow-up e-mail after the delivery of the materials for analysis; regular reminders of the tasks and deadlines; telephone and e-mail communications for clarifications, between the main researcher and the panel; revision of the answers received; and e-mail messages to provide missing ratings.

A group of 17 experts actively participated in the first round; Appendix 1 presents a table with the demographic characterization of the recruited. Having received their answers, each item under analysis (hereafter designated as indication) was evaluated as “Appropriate” (A), “Uncertain” (U) or “Inappropriate” (I), following the RAND/UCLA manual’s definitions^38^. An indication should be rated as A whenever the median of the panel’s responses is of 7–9, without disagreement between the experts. To judge the level of agreement between experts, the measure of dispersion utilized was the Interpercentile Range Adjusted for Symmetry (IPRAS), considering that it is applicable to any panel size and with good evaluations in terms of sensitivity and specificity. To calculate the IPRAS, the used formula was:

$${\text{IPRAS }} = {\text{ IPRr }} + \, \left( {{\text{AI }}*{\text{ CFA}}} \right),$$ where,

- IPRr is the Interpercentile Range required for disagreement when perfect symmetry exists, with a value of 2,35;
- AI is the Asymmetry Index; and
- CFA is the Correction Factor for Asymmetry, with a value of 1,5.

The software Microsoft Excel was used to calculate the necessary measures and all the calculations followed the RAND/UCLA’s guidelines^38^. In summary, when the IPRAS of a particular indication is smaller than the IPR of that same indication, such indication is rated with disagreement.

**Figure 4:**
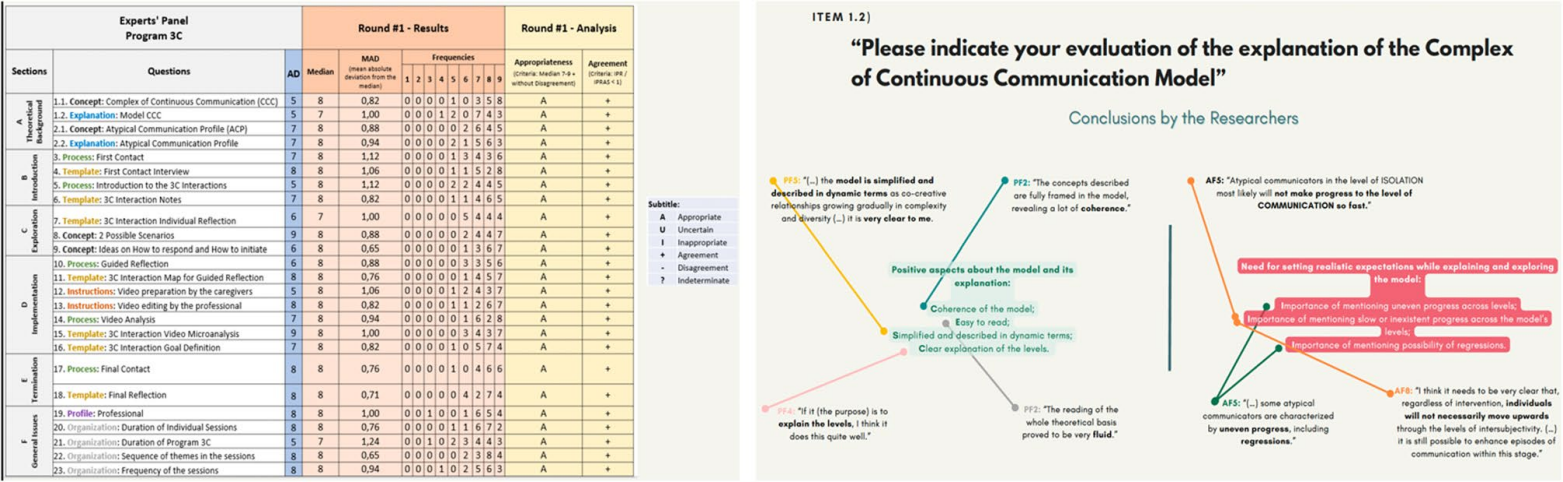
Samples of feedback for the experts after round 1—quantitative data (left) and qualitative data (right)—using the software Microsoft Excel (left) and the software Canva (right).

To follow the Appropriateness Method, the second round of the panel was held synchronously, so that the experts would have opportunity to interact, exchange ideas and adjust their rating. Prior to the second round, each participant received an Individual Rating Sheet (IRS) with own ratings and the possibility to compare those with the average, median, mean absolute deviation from the median (MAD) and frequencies of answers for each value in the rating scale. The rating in terms of appropriateness and disagreement were also presented; a sample of an IRS is presented below (Fig. 4). Even though it is not considered part of the method, a synthesis of the main findings from the experts’ qualitative feedback was shared in the IRS. This content was prepared using thematic analysis^42^, with the goal of enriching the guided discussion to be held during round number two; a sample of the qualitative feedback is presented in Fig. 4.

The second round of the panel was held synchronously with 13 participants, in December 2022, with the definition of two different dates and schedules using the online Doodle tool. Considering the participation of experts from different countries and time zones, the panel members were involved in two different online sessions using the software Zoom. The main researcher led the discussion session, using a dynamic Microsoft PowerPoint presentation and the sum of all the IRS. All indications were rated a second time, by the experts, with more detailed discussions around items with lower median value or higher MAD value. The expert’s rating was collected using the RQ, this time in an online version using Google Forms. The session as recorded and, to facilitate note taking and time management, an assistant researcher was supporting the main researcher. Finalized the second round, each indication was once again evaluated according to its Appropriateness level and Agreement between experts, using the IPRAS.

A Post-Panel Questionnaire, prepared according to the Appropriate Method manual, was sent to gather input about the experts’ experience in the panel, concerning: the review of material about the Program 3^C^; the first-round rating process; the online focus group meeting; and the overall impression of the experience. The experts’ answer was based on a five-points Likert scale, “1” being the lowest valuation and “5” being the highest. A total of nine participants responded to the post-panel enquiry, sharing high levels of satisfaction in relation to their participation in the process (average answer of 4, 56) and indicating that their participation had mostly met their expectations (average answer of 4). When asked about the role of the moderator in facilitating the live discussions, the assessment was equally positive (average answer of 4, 89), as well as in relation to how informative was the focus groups (average answer of 4, 67) and how argumentative these sessions were (average answer of 4). It became apparent throughout the process, and it was confirmed by the survey, that the review phase was not considered to be easy (average rating of 3) and was seen as an onerous task (average answer of 3, 11). Even so, additional input from the respondents enlightened that the process was taken positively by most of the participants:

- I think the process has been really worthwhile. Through the process I have gained a greater understanding of the intended audience of the program. (…) The thing that I have valued most has been the opportunity to talk with other people about their perceptions, at times having my questions validated by others, but also demonstrating a breadth of views.
- I think and believe that panels and discussion are very productive, after and before filling the different tasks.

Beforehand of any initiative by the researchers, the research project was submitted for scrutiny and validation by the Ethics Committee on Health (ECH) of the Institute of Health Sciences of the Portuguese Catholic University. Validation was granted after clarifications provided by the first author, ensuring that any experimental protocols were approved by the ECH and all methods were carried out in accordance with relevant guidelines and regulations.

## Results

Two main products resulted from the research process: (i) the Complex of Continuous Communication Model and (ii) the intervention Program 3^c^. Both are comprehensively presented over the next section.

### The complex of continuous communication model

**Figure 5:**
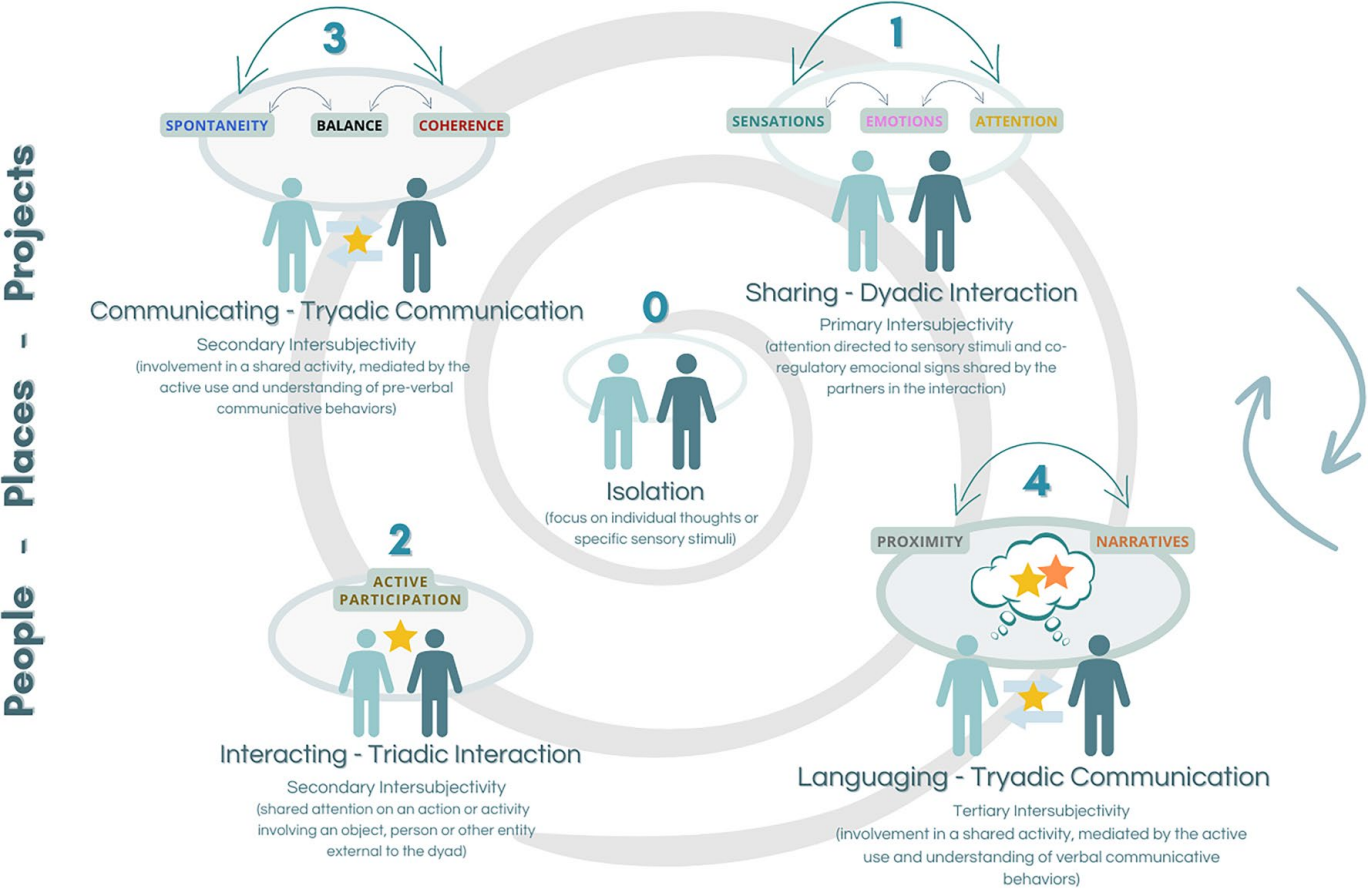
Original illustration of the Complex of Continuous Communication Model prepared by the authors using the software Canva.

As a theoretical model to illustrate how interpersonal communication emerges and evolves between people with and without CCN, the CCC rescues the omnidirectionality notion from the Transactional Model of Communication^4,5^, representing communication as a “multiple-way avenue” and presuming that the relationship between communicators is characterized by mutual influences in a process of co-creation of shared meaning. The model also incorporates the diachronic concept from the same paradigm^3^, meaning that communication is unrepeatable and may continuously “grow” in time, with the added experience from the partners; a helical shape is used in the graphical representation of the model, to display this idea. The CCC model focuses on the representation of communication complexity developing over time, representing the growing degrees of coordination between partners, and between the dyad and the context (i.e., persons, objects, or activities). It also depicts the use and understanding of increasingly sophisticated forms of communication, starting from the (undesirable) absence of initiatives/responses from the partners in isolation, to mild changes in their behaviors while in interaction with each other, triadic interactions involving elements from the context, and exchange of potentially communicative behaviors or even gestures and words^43^. The CCC model is depicted in the following image (Fig. 5).

The four levels of complexity—Sharing, Interacting, Communicating and *Languaging*—indicate different types of exchanges that may be co-created between the communication partners. As indicated by the arrows on the right, the partners may “navigate” amidst these four stages, depending on the skills, preferences and needs of the dyad; there is no demand or “pressure” to communicate at a “higher” level, if there is quality in the exchange and gratification from all parties. The three words on the left—People, Places and Project—represent the variables in the context that contribute to the meaning that is being negotiated. Across the four levels representative of interaction and communication, nine variables – Sensations, Emotions, Attention, Active Participation, Spontaneity, Balance, Coherence, Proximity and Narratives – are the fundamental components that make up the communicate exchanges co-created by the communicators, building on findings from previous research^16^.

The analysis by the experts after the second round granted validation to both indications – the model itself and its narrative explanation – as the rating by the respondents corresponded to higher values – median of eight – and there was no significant dispersion amongst the answers. The concept and explanation of Atypical Communication Profile, present in the first version of the model, were considered appropriate in the overall quantitative evaluation. Even so, the higher dispersion, the lower value of median (seven) and, most of all, the feedback from the discussion during the live round with experts supported the decision of removing these items. The main reasons for this exclusion were the attempt to reduce the use of jargon and the fact that the concept did not add much to the program’s theoretical knowledge and could be read as judgmental of the value of different communication profiles. Table 1 presents the results after the second round, with the answers from the 13 experts (E), the median values and the final rating in terms of Appropriateness and Agreement.

**Table 1:** Quantitative evaluation of appropriateness – CCC model.

Indications	Experts’ answers													Median	Appropriateness	Agreement
	Academic field						Professional practice									
	1	2	3	4	5	6	7	8	9	10	11	12	13			
Concept: CCC Model	8	9	9	8	6	7	9	8	9	9	9	8	7	8	A	+
Explanation: CCC Model	5	9	8	8	7	4	8	8	8	7	9	7	7	8	A	+
Concept: Atypical communication profile	6	7	9	7	3	5	7	8	9	7	8	7	7	7	A	+
Explanation: Atypical communication profile	5	8	6	7	3	5	7	8	9	7	8	8	7	7	A	+

A Appropriate, + Agreement.

Some positive remarks from experts about the CCC model underscore its:

(i) Coherence – as mentioned by one of the experts, “*the concepts described are fully framed in the model, revealing coherence*”.
(ii) Clarity – one expert expressed that “*if the purpose is to explain the levels, I think it does this quite well*”;
(iii) Readability – “*the reading of the whole theoretical basis proved to be very fluid*”, in one of the experts’ opinion;
(iv) Dynamism – statements like “*the model is simplified and described in dynamic terms as co-creative relationships growing gradually in complexity and diversity*”, reinforce this characteristic.

Particular improvements were made to the model, according to the discussion held during the synchronous group sessions, specifically:

(i) The elimination of the concept of Atypical Communication Profile, as it was considered potentially judgmental and not of added value;
(ii) The reconfiguration of the elliptical shape horizontally, not to use a vertical perspective which could lead to the belief that higher levels were necessarily better and needed for communication to be enriching and satisfactory for the involved;
(iii) The representation of two partners, instead of only one, in the stage of isolation, reinforcing the difference between being alone and being isolated from communication;
(iv) The use of numbers to identify each different level, to facilitate reading and interpreting the model.

Some other suggestions emanated from the analysis, such as the use of videos or case descriptions to illustrate the different levels of engagement, the importance of clarifying that there is no “right” or “wrong” level to communicate and that progression across levels will not be linear or predictable, and the interest in further exploring some concepts and nuances in the used terminology (e.g., “*refer in more detail to what is meant by joint attention*?”).

### The Program 3^C^

Regarding its structure, the finalized version of the Program 3^C^ – after the literature review and two rounds of experts – consists of an intervention with a minimum length of five moments of consultations between the professional and the caregiver(s) of the person with CCN. The minimal length of the program was defined considering the need for the participants to experience the different processes of the program: (i) First Contact Interview, (ii) introduction to the of 3^C^ interactions, (iii) implementation of the 3^C^ Guided Reflection, (iv) Video Analysis and (v) Final Contact. According to the experts’ input, the program’s implementation would come to an end as a joint decision between the professional and the caregiver(s), depending on how the intervention was meeting the needs and expectations of the last; for this reason, the maximum number of sessions was determined to be flexible. The program’s sessions are intended to be implemented on a weekly basis.

The initial session of the Program 3^C^ is devoted to use the *First Contact Interview* template to promote an important moment to gather in-depth information about the profile, expectations and needs of the caregiver(s) and person with CCN. This first exchange is also dedicated to the presentation of the program and introduction of its theoretical foundations (i.e., the CCC model). A major component of the program – the 3^C^ interactions – is explored in the introductory session, with the caregiver(s) receiving guidelines to implement regular moments of 1:1 interaction with the person with CCN being cared for.

In the first consultation (#1), the practice of the 3^C^ interactions is further explored, with the introduction of the 3^C^ “journaling” process and the presentation of the template designed for this purpose (the *3*^*C*^* Interaction Journal*). The caregivers are instructed to keep notes on the regular moments of 3^C^ interactions previously introduced and the professional takes the opportunity to present two important concepts of the intervention: (i) the three different possible interaction scenarios and how to interpret them; and (ii) ideas on how to initiate and how to respond during the 3^C^ interactions. These are merely theoretical frameworks to assist decision-making and reflection by the caregivers, about communication exchanges with the person with CCN.

With the succeeding session—Consultation #2—the goal is to elevate the reflection about the 3^C^ interactions, using the *3*^*C*^* Interaction Map for Guided Reflection* template, to support the caregiver(s) in understanding what is positive and what needs to be improved in the interaction with the person with CCN, having the different levels of complexity and the nine components of the CCC model as conceptual background. In this same session, the professional provides guidance to caregivers on how to prepare videos for the video analysis process ahead.

The following meetings constitute the core of the Program 3^C^. In each of them the specific categories and levels of engagement of the CCC model are explored, within a dialogue with the caregivers and having video analysis of high-quality communicative exchanges as "landscape" for the conversations. Distinct templates may be used to support the video analysis, and the goal definition process is introduced and practiced, using the *3*^*C*^* Interaction Goal Definition* template, to level up the quality and diversity of 3^C^ interactions. The dialogical inclination of the program inspires the co-creation of new ways of sharing, interacting, communicating and “languaging” between the caregiver(s) and the person with CCN, with the professional being expected to describe what is observed in the video footage, appraise the best communication moments, probe to support reflection by the caregiver(s) and challenge in findings new ways to diversity and enrich communication exchanges. Within this dialogue, the caregivers are expected to recognize and acknowledge strengths and points for improvement and gain new insights and ideas to explore new possibilities of communication.

**Figure 6:**
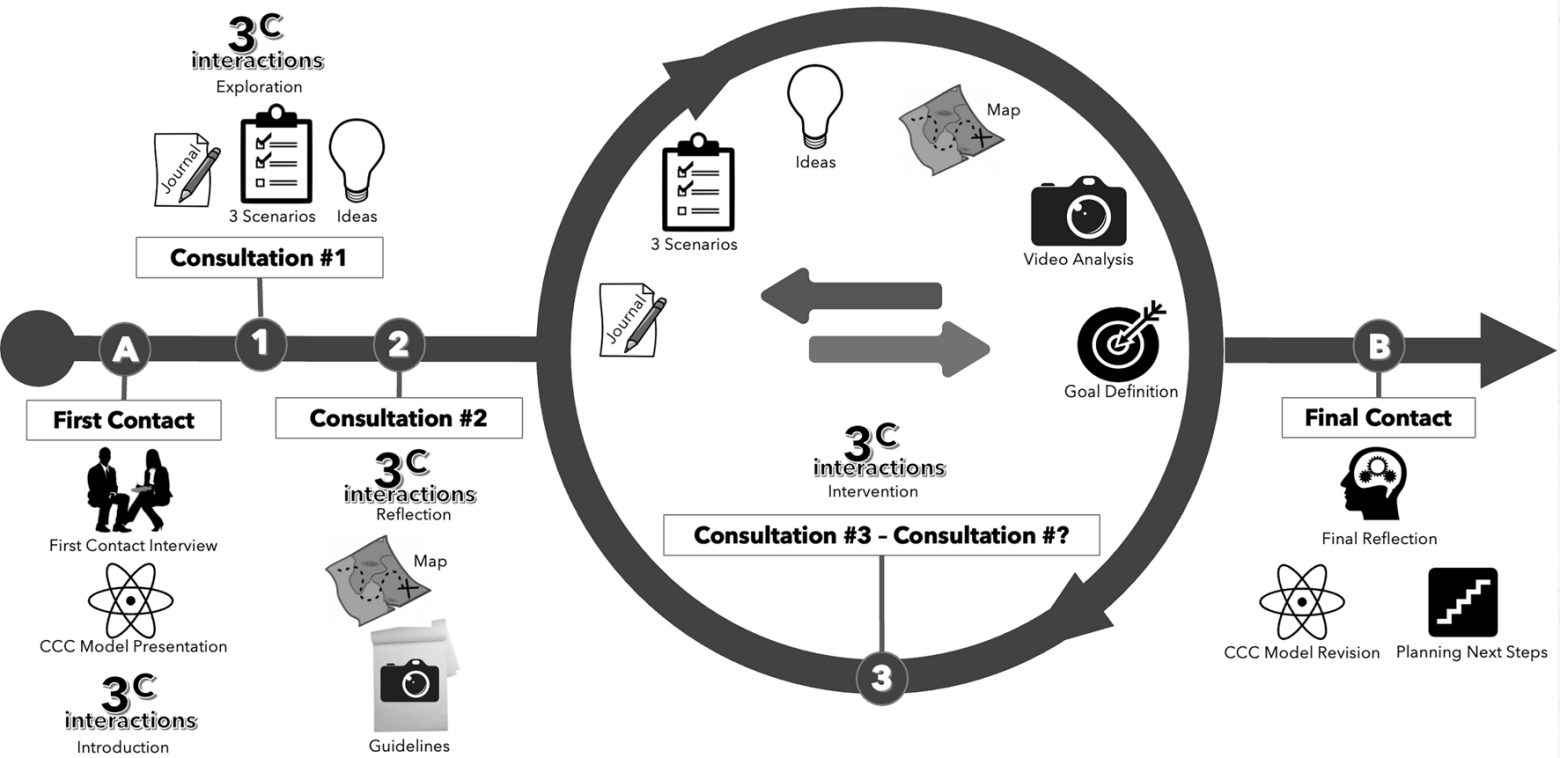
Original illustration of the Structure of the Program 3^C^ prepared by the authors using the software Microsoft Powerpoint.

The last session is devoted to reviewing the program and finalizing the intervention, with a shared decision of ending the program’s implementation, continuing with it, or moving on to alternative or supplementary approaches; for this end, the *Final Contact* template was prepared. Depending on specific needs, there is the possibility of extending the number of sessions to further address levels of involvement or components of the CCC model and/or to involve other experts to address specific topics (e.g., involving a sensory integration specialist). A graphical representation of the different phases of the program is presented below (Fig. 6).

Past the second round with the experts, all the 21 indications under analysis were rated as Appropriate, most of them with a median rating of eight. Indications referred to specific aspects of the Program 3^C^: procedures and working instructions; the concepts underlying the program; the templates being used; the profile of the practitioner; and organizational aspects around the Program 3^C^. Below, Table 2 presents the results of the quantitative evaluation after the first and second rounds with the experts, designating each indication undergoing evaluation.

**Table 2:** Quantitative evaluation of appropriateness – program 3^C^ intervention.

Indications	Experts’ answers													Median	Appropriateness	Agreement
	Academic field						Professional practice									
	1	2	3	4	5	6	7	8	9	10	11	12	13			
Process: First contact	8	9	6	5	5	6	8	7	9	8	9	8	7	8	A	+
Template: First contact interview	9	9	9	5	7	7	9	7	7	8	8	8	7	8	A	+
Process: Introduction to the 3^C^ interactions	7	9	6	5	7	7	8	8	8	8	9	7	7	7	A	+
Template: 3^C^ Interaction Journal	7	9	8	5	4	8	8	7	8	8	8	8	7	8	A	+
Template: 3^C^ Interaction Individual Reflection	7	9	6	6	5	8	7	8	6	8	8	7	7	7	A	+
Concept: 3 Possible scenarios	9	8	7	7	5	8	8	8	7	8	8	8	7	8	A	+
Concept: Ideas on how to respond and how to initiate	9	9	7	9	8	6	8	8	9	8	9	8	7	8	A	+
Process: Guided reflection	9	9	8	9	8	7	9	8	8	8	8	8	7	8	A	+
Template: 3^C^ Interaction map for guided reflection	8	9	9	9	7	7	9	8	9	8	8	9	7	8	A	+
Instructions: Video preparation by the caregivers	8	9	8	9	6	7	9	8	7	8	9	8	7	8	A	+
Instructions: Video editing by the professional	9	9	9	9	6	6	9	8	6	8	9	8	7	8	A	+
Process: Video analysis	7	9	7	9	6	6	9	8	7	8	9	9	7	8	A	+
Template: 3^C^ Interaction video microanalysis	8	9	6	9	6	6	9	7	6	8	7	9	7	7	A	+
Template: 3^C^ Interaction goal definition	8	9	7	8	6	5	9	8	8	8	8	7	7	8	A	+
Process: Final contact	9	9	5	8	5	7	8	8	9	8	8	8	7	8	A	+
Template: Final reflection	8	9	6	8	5	7	8	8	6	8	9	7	7	8	A	+
Profile: Professional	6	9	9	9	9	4	8	7	7	8	9	8	7	8	A	+
Organization: Duration of individual sessions	7	8	8	8	7	7	8	7	8	7	9	8	8	8	A	+
Organization: Duration of program 3^C^	9	8	5	3	7	7	9	7	8	7	8	9	7	7	A	+
Organization: Sequence of themes in the sessions	8	9	8	8	8	7	8	7	8	8	9	8	7	8	A	+
Organization: Frequency of the sessions	6	8	4	8	8	7	8	7	9	7	8	8	7	8	A	+

A Appropriate, + Agreement.

Several improvements were made succeeding the input from the two rounds with the experts. The main amendments made to the Program 3^C^ were:

(i) The definition of five as the minimal number of sessions for the program to be implemented, so that the professional and caregivers would have the opportunity to experience the different processes inherent to the intervention;
(ii) The designation of a flexible length of the program, without a fixed maximum number of sessions (in the proposal, a fixed number of 11 sessions was determined), being the continuation or termination of the intervention jointly defined depending on the needs and expectations of the participants;
(iii) The reduction of templates used while working with caregivers, with the elimination of the *3*^*C*^* Interaction Individual Reflection* template, thus reducing the administrative demand to be requested, since the opinion of the panel yielded that the number of notes and reflections from the first version of Program 3^C^ could be seen as a burden;
(iv) The simplification of the *3*^*C*^* Interaction Journal* template, making it more “user friendly” and less relatable to a demanding or “academic” task;
(v) The creation of a supporting template for the professional to analyze and improve collaboration with caregivers, considering ethical concerns brought in the experts’ discussion about how to deal with situations in which there would not be a productive collaboration process;
(vi) The redesigning of some visual supports presented to the caregivers, to better depict the transactional and dialogical nature of the intervention and enhance the relevance of trying to incorporate input from the person with CCN in the dialogues to enrich communication exchanges.

Although opportunities for improvements were evident, the program benefited from overall acceptance from the panel, with positive remarks emanating from the thematic analysis. By way of illustration, the feedback from three experts is transcribed:

“The program you offer is significant and relevant to many people with communication difficulties. One can see how much thought has been invested in building the program and how much it rests on theoretical foundations. The idea of supporting caregivers over time while they experience interaction events with atypical communicators is excellent.”;

“I think the strengths of this program relies in the fact that it is continuous (not just one or two training sessions) and requires caregivers to be active learners. It is good practice to base it on theory – I would anticipate that discussions would cover a wider scope in practice.”;

“Very interesting to use dialogue as a strategy for training sessions”.

Additional deliberations were made and are left as input to be considered during the first pilot study using the program, for instance: the value of implementing the programs with groups of caregivers, to promote peer-support and reduce the pressure of a 1:1 intervention; the need to ensure professionals have enough background knowledge on the topic of CCN; and the concern that the individual implementation of the proposed intervention may be considered expensive by the service provider.

## Discussion

The goal of this investigation was to co-create a conceptual model to explain and improve interpersonal communication between caregivers and their partners with CCN. The CCC, as a conceptual model to analyze atypical communication, represents different levels of complexity in interaction/communication, with a simple terminology based on caregivers’ vocabulary and illustrated by a clear graphical representation. A second objective was to propose a dynamic and responsive intervention program, based on the new model, to support communication interventionists, namely SLT, in their work supporting caregivers. Through the validation of the suggested model and program with an experts’ panel, the team considers both research goals to have been achieved.

According to recent research^44^, there is a paucity of methods to conduct a detailed analysis of communication behaviors of individuals with CCN. This gap challenges researchers and clinicians to develop novel models and approaches, reinforcing the added value of the present project. As expressed by the experts, the model shows strengths in terms of being a dynamic, clear and coherent explanation of different levels of interaction and communication, passible to be used with caregivers of persons with CCN. Nevertheless, the enquiry with the experts emphasized opportunities for improving the model:

1. One panelist mentioned that “*in a communication process in which one partner has a disability, both partners become communicatively disabled*.” It is, therefore, reasonable to conclude that it matters to reinforce this notion of shared responsibility, when it comes to effective communication, as it was theoretically acknowledged previously in this text^14,16,23^ and aligned with the biopsychosocial conceptualization of disability^45^.
2. The CCC model anchors part of the analysis in the concept of communicative intentionality and this was pointed out as an aspect worthy of reflection. The input from one respondent emphasized that “*historically (…) we've been too eager to say it* – a communicative behavior – *is not partner directed, partly because it fails to meet particular behavioral requirements*”. Referring to Carter and Iacono’s work^46^, there is meaningful inconsistency in how professionals and researchers judge the intentionality of communicative attempts of “early communicators”, making it fundamental for this judgement to be based on strong theoretical archetypes and unbiassed and published clinical criteria.
3. Related to the previous point and following the dialogical orientation of the model, one expert suggested to “*reserve judgement of the intention of the communicator but accept the pragmatic function possibilities/interpretations/negotiations*”. This is the perspective to consider when adopting the CCC model, which might not be entirely clear in the proposal.
4. Lastly, it is accepted that the model shows the fragility of not representing in detail the context in which communication takes place, as stated by one panel participant. While the importance of taking an ecological and activity-based approach in the intervention with persons with severe disability is widely known and recognized^47^, this model “only” refers to the persons, places, and projects (i.e., actions, activities) in the context. It does not, however, entail comprehensive features of the setting, as other contemporary models do^44^. It was the option of the authors to focus the analysis on the growing communicative relationship between partners, with no intent of lessening the importance of other foci and without disregarding the use of other models and approaches to include different dimensions in the analysis.

As for the Program 3^C^, it was found that it follows a structure comparable to other validated intervention proposals^48–51^ in terms of the type of participants, number and scheduling of the sessions, integration of theoretical component parallel to a practical implementation of strategies, and use of video analysis as a working process. To work with caregivers with such approach appears to be a promising practice, as expressed by one of the participants in the panel: “*the focus on empowering the role of parents/caretakers as communication partners is really needed and highly appropriate (…) it is important they have options in time to be really engaged and understand the model (…) in my experience, understanding the conceptual structure in an intervention model can really help them interact/communicate with their child*”. In order to work in the area of communication with relatives of children with multiple disabilities, Flink, Johnels, Broberg and Thunberg^52^ created a training program consisting of eight sessions, incorporating a balanced theoretical and practical component to explore topics such as communication, characteristics of good communication partners, introduction to augmentative communication, among others. This intervention, tested with several families, was positively evaluated, not only for the learning achieved, but also for the peer support within the group, echoing some of the positive aspects of Program 3^C^. Even though the appropriateness and agreement score were very positive, specific improvements were made to the Program 3^C^, as explored in a previous section of the paper.

Lastly, it matters to focus an important concern raised by one of the experts: “*What is the role of your focused person* – i.e., the person with CCN – *in this co-creative process?* (…) *Do they get a role? Do they get an input? Should they feature here*?”. To “give voice” to the person with CCN during the intervention should be a major concern in a program like the Program 3^C^. Some suggestions surfaced from the group dialogue:

- Whenever possible, to directly ask the person with CCN about the preferred moments of communication and engagement;
- To use storytelling to share about the communication experiences under analysis;
- To consider that the video footage under analysis, where the person with CCN is featured, is a way to bring his contribution into the dialogue.

Having in mind that the target population are persons with complex disability (e.g., severe autism, profound intellectual and multiple disability, congenital deafblindness), the group concluded that additional measures would be challenging in many of the cases. Voiced by one of the experts: “*it’s good in principle to make a space for that individual to have some input if that’s possible*”. Having this in mind, the visual representation of the dialogical process of video analysis was reviewed to ensure the person with CCN is represented and as a reminder of bring his contribution into the dialogue whenever and however that is possible.

It matters to acknowledge the crucial role of the caregivers and how the positive outcome of the intervention depends on them. This was valued during the experts’ discussions—“*The idea of supporting caregivers over time while they experience interaction events with atypical communicators is excellent*”. It does ask from the professionals to presume competence and give significance to the input of caregivers while analyzing communication exchanges and making decisions on how to enhance them. Though at times the information given by caregivers may seem like intuitive or from a “sixth sense”, research has shown that the parents’ knowledge may be crucial in the understanding improving care for people with multiple disability^53^. The argument of “presuming competence” has for long been object of reflection by professionals, when it comes to acknowledge the perspective of persons with a disability^54^ and to adopt inclusive approaches in the field of communication^55^. Let the Program 3^C^, anchored in a transactional understanding of communication and inspired by the dialogical philosophy, be a resource center to work with caregivers, presuming they may be(come) the most competent of communication partners for whom look after. When questioned about the importance of a genuine cooperation with professionals, parents identified seven elements for the ideal partnership, including the professional’s preparation to work with persons with disability and their expertise on the topic being covered^56^. With its clear structure and flexible features, it is plausible to assume that the Program 3^C^ may support communication specialists building their capacity to work with caregivers and contributing to their expertise in the field of communication processes and intrinsic complications and barriers.

Lastly, the option for using the DM to develop an expert-based judgment is based on positive results from previous research, and based on the assumption that the multitude of perspectives within a group of experts will produce valid results, especially when the subject under study involves subjective expertise and has a complex and multidisciplinary nature^35,57^. The use of modified and online Delphi Panels offers a cost-effective and convenient alternative to the traditional in-person modality. Even though it has been widely used in Health research, its diversified utilization calls for caution and requires the application of high-quality assurance criteria to guarantee reliable findings^40^. Aware of this limitation, specific recommendations from the literature^38,40^ have been followed, explicitly: using validated reporting templates from the RAND/UCLA manual; establishing measures of process quality, through a quality evaluation questionnaire for the experts; and utilizing an online platform that would allow the engagement, interaction and sharing among the panelists. Additional precautions were taken with the recruitment of experts to ensure cognitive diversity, by involving participants with diverse backgrounds and from different countries, as well as with the option for a clear definition and quantitative measure to outline consensus, for the sake of the transparency of the results^57^.

### Supplementary Information

Supplementary Information.

## Data Availability

The data generated and analyzed during this study is included in this published article and its supplementary information files. Additional raw datasets analyzed during the current study may be made available from the corresponding author on reasonable request.
