# Supplementary material for: A new intervention to prevent social isolation in people with complex communication needs
Source: Sci Rep. 2024 Jun 3;14:12742. doi: 10.1038/s41598-024-63519-5 (PMC11148034; doi:10.1038/s41598-024-63519-5)
Supplement: Supplementary file 1 — Supplementary Information. [file 41598_2024_63519_MOESM1_ESM.docx]

# Appendix I – Characterization of the Group of Experts

| **Experts** | ***Academia*** | | | | | | | |
| --- | --- | --- | --- | --- | --- | --- | --- | --- |
|  | **EA1** | **EA2** | **EA3** | **EA4** | **EA5** | **EA6** | **EA7** | **EA8** |
| **Age** | 42 years | 71 years | 51 years | 62 years | 73 years | 68 years | 60 years | 46 years |
| **Gender** | Female | Female | Female | Female | Female | Female | Female | Female |
| **Nationality** | Belgian | Portuguese | British | Brazilian | British | Israeli | Israeli | Australian |
| **Profession** | Lecturer and Researcher | SLT  Lecturer | SLT  Lecturer | SLT  Lecturer | SLT | SLT  Lecturer | SLT  Lecturer | SLT |
| **Education** | Ph.D. | Ph.D. | Ph.D. | Ph.D. | Ph.D. | Ph.D. | Ph.D. | Ph.D. |
| **Professional experience in the field of CCN** | 6 years | 30 years | 27 years | 40 years | 38 years | over 30 years | over 25 years | 24 years |
| **Academic experience in the field of CCN** | 9 years | 20 years | 8 years | 19 years | 30 years | over 20 years | over 25 years | 15 years |

| **Experts** | **Professional Field** | | | | | | | | |
| --- | --- | --- | --- | --- | --- | --- | --- | --- | --- |
|  | **EP1** | **EP2** | **EP3** | **EP4** | **EP5** | **EP6** | **EP7** | **EP8** | **EP9** |
| **Age** | 40 years | 37 years | 46 years | 48 years | 73 years | 68 years | 35 years | 46 years | 49 years |
| **Gender** | Female | Female | Female | Female | Female | Female | Male | Female | Female |
| **Nationality** | Portuguese | Portuguese | Portuguese | American | British | Norwegian | Chilean | Dutch | Dutch |
| **Profession** | SLT  Lecturer | SLT | SLT  Lecturer | SLT  Trainer | SLT | Psychologist  Lecturer | SLT | Occupational Therapist | Occupational Therapist |
| **Education** | M.Sc. | M.Sc. | Specialist Certificate | M.Sc. | Ph.D. | Ph.D. Candidate | Graduate | M.Sc. Candidate | Graduate |
| **Professional experience in the field of CCN** | 18 years | 16 years | 25 years | 25 years | 38 years | 40 years | 10 years | 28 years | 25 years |
| **Academic experience in the field of CCN** | 15 years | --- | 23 years | 2 years | 30 years | 20 years | 5 years | --- | 2 years |
